# Education-Related Entrepreneurial Competence, Resource-Opportunity Accessibility, Self-Efficacy, and Entrepreneurial Intention: Evidence from a Vocational College in Yiwu, China

**DOI:** 10.3390/bs16091568

**Published:** 2026-09-04

**Authors:** Feng Lili, Kenny S. L. Cheah, Serrene Leong

**Affiliations:** 1 Institute for Advanced Studies, Universiti Malaya, Kuala Lumpur 50603, Malaysia; 17220958@siswaold.um.edu.my; 2 Faculty of Education, Universiti Malaya, Kuala Lumpur 50603, Malaysia; 3 Faculty of Business and Economics, Universiti Malaya, Kuala Lumpur 50603, Malaysia; serrene@um.edu.my

**Keywords:** education-related entrepreneurial competence, entrepreneurial self-efficacy, entrepreneurial intention, resource-opportunity accessibility proxy, vocational education

## Abstract

Entrepreneurship education (EE) is often linked to entrepreneurial intention (EI) through entrepreneurial self-efficacy (ESE), but resource-rich vocational settings may also shape whether students perceive resources and opportunities as accessible. Using survey data from 322 vocational students in Yiwu, China, this cross-sectional single-institution study estimates a partial Social Cognitive Career Theory (SCCT)-informed composite model. The model distinguishes an education-related entrepreneurial competence core (EE-core), a competence-based resource-opportunity accessibility (ROA) proxy, ESE, and EI. EE-core was positively associated with ESE (β = 0.257, *p* < 0.001), and ESE with EI (β = 0.257, *p* < 0.001). The bootstrapped EE-core-ESE-EI indirect association was positive but small (β = 0.066, 95% CI [0.025, 0.121]). ROA was also positively associated with EI after accounting for EE-core and ESE (β = 0.150, *p* = 0.028) and remained positive after controlling for gender and grade (β = 0.143, *p* = 0.039). An exploratory bootstrap contrast showed no clear difference between the direct ROA estimate and the EE-core-ESE-EI indirect estimate. The EI model explained 12.9% of variance. Findings should be interpreted cautiously because ROA is a competence-based proxy and the data are cross-sectional, self-reported, and drawn from one institution.

## 1. Introduction

Entrepreneurship education (EE) is widely used to develop students’ entrepreneurial awareness, skills, and career readiness. This role is especially salient in vocational colleges, where education is expected to connect classroom learning with applied work, local industry participation, and practice-based innovation. Prior research links EE to entrepreneurial intention (EI), often through internal psychological mechanisms such as entrepreneurial self-efficacy (ESE) (Martin et al., 2013; Nabi et al., 2017; Piperopoulos & Dimov, 2015; Wu et al., 2022). Yet this emphasis is incomplete when students learn in settings where entrepreneurial resources, market channels, and role models are highly visible. Recent evidence similarly suggests that EE often works through capability beliefs, psychological resources, and educational conditions rather than through simple direct exposure alone (X.-H. Wang et al., 2023).

The argument here is not that entrepreneurship is the best or only pathway for vocational students. EI is examined as one career-choice goal within a wider career-readiness agenda. In practice-oriented economies, the boundaries among employment, self-employment, platform work, family or micro-enterprise participation, and small venture creation can be fluid. EE may therefore matter not only by encouraging firm creation, but also by developing opportunity recognition, resource mobilization, uncertainty handling, and market-facing capabilities across vocational career paths.

Yiwu, China, provides a useful setting for examining this issue. The city is known internationally for its small-commodity market, dense supplier networks, logistics capacity, digital commerce channels, and cross-border trade infrastructure (W. Liu & Si, 2022; Qian et al., 2024; Q. Wang et al., 2019; Wei et al., 2020). For vocational students, entrepreneurship is not only an abstract career ideal; it is a visible local practice linked to markets, platforms, mentors, products, and school-enterprise cooperation. This context makes Yiwu suitable for examining whether students’ EI is associated not only with confidence in entrepreneurial tasks, but also with perceived access to concrete resources and opportunities. Because the study includes one institution and no comparison site, Yiwu is treated as a bounded context rather than as a tested moderator.

A persistent problem in EE research is that education-related competence, contextual support, and self-efficacy pathways are sometimes conflated in intention models. This creates a construct-overlap risk: a subdomain of education-related competence should not be treated as a fully independent environmental construct unless it has been measured and validated separately. To avoid double counting item content, the present analysis uses non-overlapping composites and labels the opportunity-identification/resource-acquisition component a resource-opportunity accessibility (ROA) proxy. ROA is a competence-based indicator that may partly reflect perceived contextual accessibility; it is not a validated stand-alone environmental-support construct.

The model is grounded in Social Cognitive Career Theory (SCCT), which links learning experiences, self-efficacy, outcome expectations, contextual supports and barriers, and choice goals (Lent & Brown, 2019; Lent et al., 1994, 2000). Within this partial framework, EE-core is treated as a learning-related competence outcome, ESE captures students’ beliefs about their ability to perform entrepreneurial tasks, ROA cautiously serves as a competence-based proxy for perceived contextual accessibility, and EI represents a choice goal. Outcome expectations, another central SCCT component, were not directly measured and therefore could not be modeled separately; this is a study boundary, not a claim that anticipated outcomes are unimportant. The Theory of Planned Behavior (TPB) remains a useful background model of intention formation (Ajzen, 1991; Krueger et al., 2000), but SCCT better fits the present focus on perceived competence, self-efficacy, contextual accessibility, and career choice.

The study addresses two substantive questions: how EE-core perceived competence, ESE, and the ROA proxy are associated with EI among vocational students in Yiwu, and whether ROA accounts for additional variance in EI after EE-core and ESE are considered. These relationships are formalized in H1-H4. Existing theory, however, does not provide a sufficiently strong basis for predicting whether the direct ROA association should be larger than the EE-core-ESE-EI indirect association. That comparison is therefore treated as an exploratory bootstrap contrast rather than as a formal research question or hypothesis.

The study makes a measurement-focused contribution to entrepreneurship-education research by applying a partial SCCT lens to a resource-rich vocational setting. The analysis separates the entrepreneurial competence scale into non-overlapping EE-core and ROA composites, examines ESE alongside a competence-based contextual-accessibility proxy, and treats Yiwu as a bounded setting for future comparison rather than as a tested moderator. This specification is intended to reduce item double counting and to distinguish the theory-specified hypotheses from the exploratory direct-versus-indirect comparison.

### 1.1. Entrepreneurship Education and Perceived Competence Outcomes

EE is intended to build knowledge, skills, motivation, and readiness for entrepreneurial action. Reviews and meta-analyses show that EE can influence entrepreneurial attitudes, intentions, and human-capital outcomes, although the size and durability of these effects vary by program design, pedagogy, evaluation design, and context (Cui et al., 2021; Fayolle & Gailly, 2015; Hägg & Gabrielsson, 2020; Longva & Foss, 2018; Martin et al., 2013; Nabi et al., 2017; Pittaway & Cope, 2007). For vocational institutions, practice-based EE is especially relevant because it emphasizes doing, market contact, skill application, and problem solving (Boldureanu et al., 2020; Lyu et al., 2024).

The evidence on EE is not uniform. Some studies report positive associations between EE and EI, whereas meta-analytic and quasi-experimental work indicates that effects can be small, heterogeneous, or contingent on prior intention, pedagogy, evaluation design, and institutional context (Bae et al., 2014; Longva & Foss, 2018; Oosterbeek et al., 2010; Rauch & Hulsink, 2015). This debate cautions against treating EE as a single undifferentiated intervention. A course focused mainly on general knowledge is unlikely to operate in the same way as a practice-based program embedded in a local market ecosystem.

Vocational education adds another boundary condition. Much EE research focuses on general university students, business students, or mixed higher-education samples. Vocational colleges differ because they emphasize applied skills, work-based learning, industry interfaces, and transitions into local labor markets. In these settings, EE may function less as abstract venture-creation advocacy and more as market-facing career preparation. Research on technical and practice-oriented education therefore suggests that EE should be evaluated through the fit among pedagogy, regional industry structure, and students’ realistic career options (Hassan et al., 2021; Lv et al., 2022; Lyu et al., 2024; Walter et al., 2013; Wardana et al., 2020).

In this study, EE is not measured as course exposure or instructional hours. The available scale developed by Yang et al. (2014) measures students perceived entrepreneurial competence, including leadership and management, strategic decision-making, innovation and creativity, pressure bearing, opportunity identification, and resource acquisition. To align the statistical model with the conceptual claim, the analysis uses the term EE-core for the non-overlapping perceived competence outcomes that do not directly represent opportunity or resource access: leadership and management, strategic decision-making, innovation and creativity, and pressure bearing. Accordingly, EE-core should be interpreted as an education-related perceived competence outcome rather than as a causal estimate of an EE intervention.

### 1.2. EE-Core Perceived Competence and Entrepreneurial Self-Efficacy

Self-efficacy refers to people’s beliefs about their capability to organize and execute actions required to attain desired outcomes (Bandura, 1997). Entrepreneurial self-efficacy (ESE) applies this logic to entrepreneurial tasks such as developing products and markets, building relationships, defining a core purpose, coping with unexpected challenges, and creating innovative environments (De Noble et al., 1999). EE-core and ESE are therefore related but conceptually distinct: EE-core summarizes students’ perceived competence-related outcomes in leadership and management, strategic decision-making, innovation and creativity, and pressure bearing, whereas ESE captures confidence in performing entrepreneurial tasks. Keeping these constructs separated is important because perceived competence content should not be treated as identical to a self-efficacy belief.

SCCT treats efficacy beliefs as one mechanism linking learning experiences with interests, goals, and choices (Lent & Brown, 2019; Lent et al., 1994). In entrepreneurship education, applied learning may provide opportunities for mastery, observation, feedback, and social persuasion that inform efficacy beliefs. Empirical studies across student samples likewise report positive associations between education- or competence-related development and ESE (X. Liu et al., 2019; X.-H. Wang et al., 2023; Wardana et al., 2020; Wu et al., 2022). Although these studies differ in how they operationalize education and competence, they converge on the same directional expectation: students who report stronger competence-related outcomes should also report greater confidence in performing entrepreneurial tasks.

**H1.**  *EE-core perceived competence is positively associated with ESE.*

### 1.3. Entrepreneurial Self-Efficacy and Entrepreneurial Intention

Entrepreneurial self-efficacy is consistently associated with entrepreneurial intention because students who believe they can perform entrepreneurial tasks are more likely to regard entrepreneurship as feasible and actionable. A systematic review and student-focused empirical studies report positive ESE-EI relationships across a range of educational settings (X. Liu et al., 2019; Newman et al., 2019; Piperopoulos & Dimov, 2015; Shahab et al., 2019; X.-H. Wang et al., 2023; Wu et al., 2022). Within SCCT, this pattern is consistent with the role of efficacy beliefs in shaping career-related goals: stronger confidence in performing relevant tasks should be associated with a stronger intention to pursue the corresponding career option (Lent & Brown, 2019; Lent et al., 1994).

Confidence alone cannot explain entrepreneurial intention because contextual supports and barriers may also matter. Even so, ESE remains a proximal belief about whether entrepreneurial tasks are within a student’s perceived capability and, therefore, whether entrepreneurship appears feasible (Saeed et al., 2015).

**H2.**  *ESE is positively associated with EI.*

### 1.4. The Indirect EE-Core-ESE-EI Association

Together, the arguments for H1 and H2 suggest a positive indirect association from EE-core perceived competence to EI through ESE. Entrepreneurship-education research has often treated ESE as an explanatory link between education-related experiences or learning outcomes and entrepreneurial intention (Piperopoulos & Dimov, 2015; X.-H. Wang et al., 2023; Wu et al., 2022). A plausible sequence is that students who report stronger competence-related outcomes also report stronger efficacy beliefs, which are in turn associated with stronger entrepreneurial intentions.

The present cross-sectional design cannot establish that this sequence unfolds over time because EE-core, ESE, and EI were measured from the same respondents on one occasion. H3 is therefore framed deliberately as a statistical indirect association rather than as evidence of temporal or causal mediation. The bootstrap test evaluates whether the product of the two component associations differs from zero without implying causal ordering (Preacher & Hayes, 2008).

**H3.**  *EE-core shows a positive indirect association with EI through ESE.*

### 1.5. Resource-Opportunity Accessibility and Entrepreneurial Intention

SCCT also emphasizes contextual supports and barriers that shape whether individuals view a career goal as reachable and sustainable (Lent & Brown, 2019; Lent et al., 2000). In entrepreneurship, relevant supports can include access to suppliers, customers, mentors, funding information, digital platforms, incubation spaces, and examples of viable ventures. Entrepreneurial-ecosystem research similarly emphasizes that networks, institutions, support services, finance, human capital, and market access condition the practical feasibility of entrepreneurial action (Belitski & Heron, 2017; Brown & Mason, 2017; Spigel, 2017; Stam, 2015).

Student-focused evidence is consistent with this contextual logic. Perceived university support and environmental characteristics have been linked with entrepreneurial intention and with the translation of intention into entrepreneurial action, suggesting that students’ goals are shaped not only by what they believe they can do but also by whether relevant resources and support appear accessible (Elnadi & Gheith, 2021; Saeed et al., 2015; Shirokova et al., 2016; Walter et al., 2013). Opportunity-identification research further suggests that entrepreneurship education can influence how students notice and evaluate entrepreneurial possibilities (Karimi et al., 2016).

Yiwu provides a bounded setting in which these accessibility perceptions are especially salient. Students are surrounded by wholesale markets, supply-chain services, digital commerce, cross-border trade, and vocational programs connected to local industries. These features do not establish a contextual effect because the study has no comparison site, but they make it theoretically plausible that students’ judgments about whether resources and opportunities are accessible may be associated with EI even after ESE is considered.

Resource-opportunity accessibility is therefore treated cautiously as a competence-based contextual-accessibility proxy. The ROA indicators come from the opportunity-identification and resource-acquisition dimensions of the entrepreneurial competence scale, so they directly measure perceived capability rather than the objective availability or quality of environmental support. They cannot, therefore, be read as a direct measure of contextual support. In a resource-rich setting, however, higher self-ratings on identifying opportunities and acquiring resources may partly reflect whether students see external resources as accessible and actionable. The contextual-support literature provides a rationale for expecting such accessibility content to matter for EI, but it does not convert ROA into a validated environmental-support construct.

**Figure 1:**
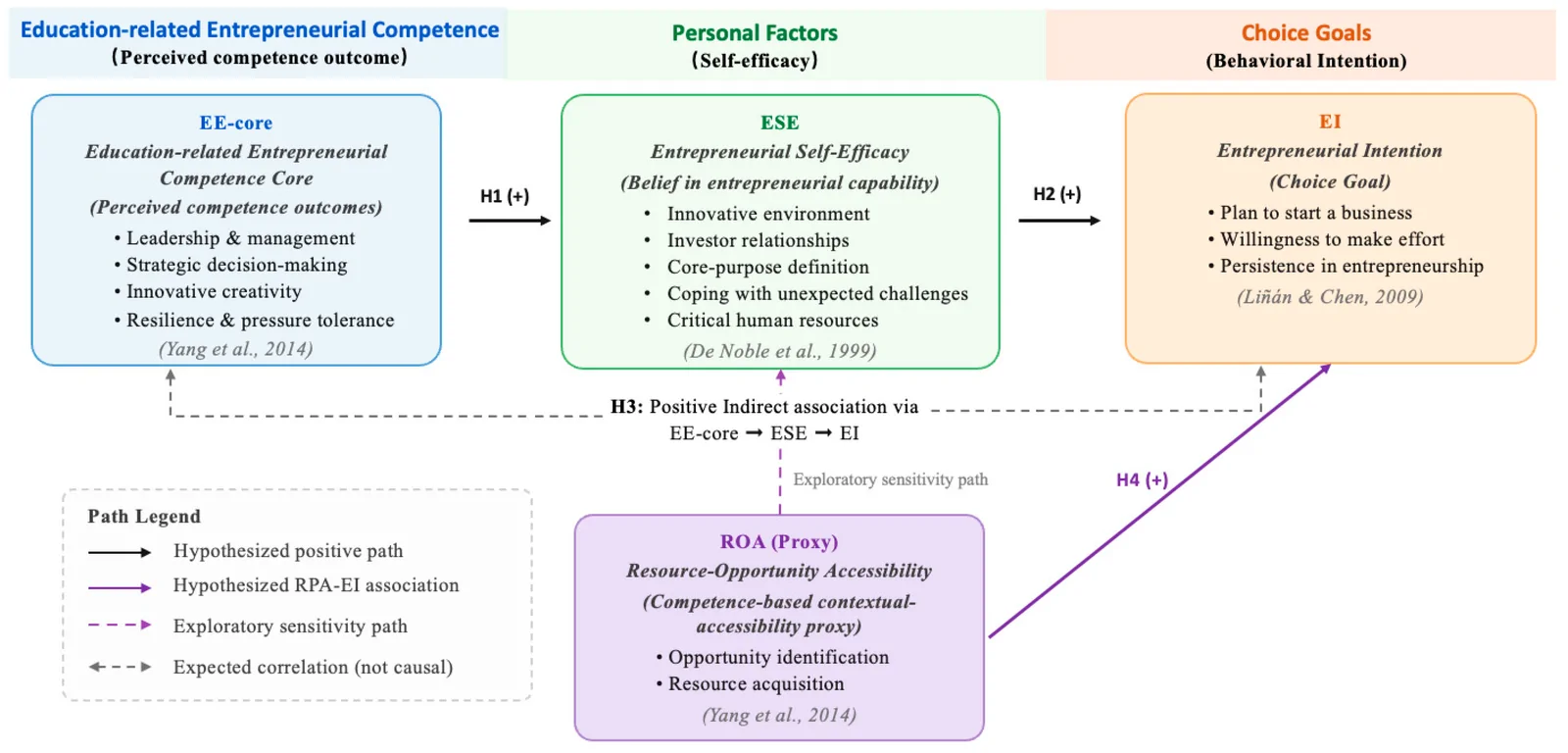
Partial SCCT-informed conceptual model of the hypothesized associations and exploratory sensitivity path. Sources for construct measures: (Yang et al., 2014), (De Noble et al., 1999), and (Liñán & Chen, 2009).

Because H4 concerns the incremental association of the ROA proxy rather than a claim that ROA is an independent environmental construct, the hypothesis is evaluated after EE-core and ESE are entered in the EI model. Thank you. We have checked and revised the text so that Table 1 and Figure 1 are first cited at the appropriate location and in numerical order.

**H4.**  *The ROA proxy is positively associated with EI after accounting for EE-core and ESE.*

## 2. Materials and Methods

### 2.1. Study Context

The study was conducted at Yiwu Industrial and Commercial College, the only higher vocational institution located in Yiwu. Yiwu was selected as a purposeful setting rather than a convenience site. The city is a resource-dense commercial ecosystem with a globally connected small-commodity market, logistics infrastructure, digital commerce platforms, and cross-border trade connections (W. Liu & Si, 2022; Qian et al., 2024; Q. Wang et al., 2019). These characteristics make the setting suitable for examining how education-related perceived competence, entrepreneurial self-efficacy, and perceived resource accessibility are associated with EI. The single-site design cannot estimate a Yiwu effect or test ecosystem moderation.

The institutional setting is also relevant. The college established an Entrepreneurship College in 2008 and has developed practice-oriented EE through e-commerce, cross-border e-commerce, live-streaming commerce, school-enterprise cooperation, campus-store practice, incubation space, and local practice bases. These details are used to contextualize the quantitative model; the hypothesis tests themselves are based on student survey data.

### 2.2. Participants and Procedure

The target population comprised students in the Entrepreneurship College of Yiwu Industrial and Commercial College. The sampling frame included 2167 students across three grades: 698 first-year students, 753 second-year students, and 716 third-year students. A stratified sampling plan preserved the grade distribution. Following the Krejcie and Morgan (1970) sample-size logic used in the doctoral project, the planned analytic sample was 322 students, allocated as 104 first-year, 112 second-year students, and 106 third-year students. Four hundred questionnaires were distributed across the three strata to allow for nonresponse and exclusion.

After records with missing responses or clearly non-attentive patterns were excluded, the analytic file retained 322 cases, equal to 80.5% of the 400 distributed questionnaires. Archived records do not retain grade-specific return and exclusion counts, so stratum-specific response rates cannot be reconstructed. The final sample included 153 male students (47.52%) and 169 female students (52.48%). By grade, 104 participants were first-year students (32.30%), 112 were second-year students (34.78%), and 106 were third-year students (32.92%).

The sample is not claimed to represent all Chinese vocational students. Its coverage is limited to the defined institutional sampling frame and the three grade strata in the Entrepreneurship College. The analysis therefore focuses on within-sample associations rather than broad population-level generalization.

A pilot stage preceded the main survey. The questionnaire was piloted with 30 vocational students in Jinhua to assess clarity, wording, and practical administration. Feedback from the pilot informed wording and sequencing before the main data collection. Participation was voluntary, informed consent was obtained, and responses were anonymized. The pilot indicated that the questionnaire was comprehensible but did not constitute a formal expert-panel content-validity assessment.

### 2.3. Measures

All focal variables were measured with seven-point Likert-type items. The instruments were translated and adapted for the Chinese vocational context through a translation and review procedure consistent with cross-cultural questionnaire practice (Brislin, 1970). Higher scores indicate higher levels of the corresponding construct. Content coverage was inherited primarily from the established instruments and supported by translation/review and the 30-student pilot; no formal expert-panel content-validity index or content-validity ratio was available.

EE-core perceived competence was derived from Yang et al.’s (2014) entrepreneurial competence scale. To avoid overlap with ROA, the EE-core composite includes leadership and management, strategic decision-making, innovation and creativity, and pressure bearing (14 items; α = 0.871, McDonald’s ω = 0.874). EE-core represents an education-related perceived competence outcome, not direct exposure to a particular EE course and not ESE.

ROA was operationalized using the opportunity-identification and resource-acquisition dimensions of Yang et al.’s (2014) scale (eight items; α = 0.846, ω = 0.848). The items assess perceived capability in identifying opportunities and acquiring or organizing resources. In Yiwu, such ratings may partly reflect whether ecosystem resources seem accessible and usable. Because the scale was not designed to measure environmental support, ROA remains a competence-based contextual-accessibility proxy.

ESE was measured with De Noble et al.’s (1999) 23-item scale (α = 0.921, ω = 0.924). The items cover students’ confidence in developing new products and markets, creating an innovative environment, building investor relationships, defining a core purpose, coping with unexpected challenges, and developing critical human resources. ESE is a capability belief, not demonstrated competence.

EI was measured using the five intention items from Liñán and Chen’s (2009) Entrepreneurial Intention Questionnaire (α = 0.897, ω = 0.900). The full 19-item EIQ was not used as the dependent variable because it also includes personal attitude, subjective norm, and perceived behavioral control, which are antecedent constructs in TPB. A robustness analysis using the full EIQ domains is reported only as a sensitivity check.

### 2.4. Composite-Score OLS Path Analysis and Robustness Procedures

The analysis was designed to address construct overlap directly. Rather than estimating a model in which a broad EE factor and its resource-opportunity subdimensions both enter the same structural path, the analysis used non-overlapping observed composites so that opportunity-identification and resource-acquisition item content was not counted in two focal predictors. The direct ROA estimate versus the EE-core-ESE-EI indirect estimate was examined only as an exploratory bootstrap contrast because the literature did not justify a directional hypothesis about which route should be larger.

All focal scores were reconstructed from respondent-level items rather than archived broad total-score columns. Two legacy broad-score columns contained discrepancies and were excluded; no corresponding inconsistencies were identified in the item responses used for the reconstructed scores. EE-core averaged four non-overlapping subdomain means, ROA averaged two, ESE averaged six, and EI was the mean of the five intention items. Scores were standardized only after composite construction and only for regression, making the scoring rules explicit and reproducible from the respondent-level item data.

Statistical analyses were conducted using IBM SPSS 26.0, with confirmatory factor analysis conducted using IBM SPSS Amos 28.0. The analysis proceeded in six steps. First, descriptive statistics, Cronbach’s α, and McDonald’s ω were calculated to summarize scale performance and internal consistency (Dunn et al., 2014). Second, Pearson and Spearman correlations and item-level heterotrait-monotrait ratios (HTMT) were examined to describe bivariate associations and assess discriminant validity; the 0.85 HTMT reference was used as a conservative diagnostic benchmark rather than as a stand-alone validity decision rule (Henseler et al., 2015). Third, standardized ordinary least squares (OLS) path regressions tested the focal associations. Fourth, 10,000 case-level bootstrap resamples estimated percentile confidence intervals for the indirect association and the exploratory direct-versus-indirect contrast (Preacher & Hayes, 2008). Fifth, HC3 heteroskedasticity-consistent standard errors assessed inferential robustness (MacKinnon & White, 1985). Sixth, predictor VIF, full collinearity VIF, Harman’s single-factor result, and one-factor versus multi-construct CFA diagnostics assessed collinearity and common method concerns (Kock, 2015; Podsakoff et al., 2003).

The primary analysis used OLS regression with standardized multi-item aggregate scores. Absolute skewness did not exceed 0.310 and absolute excess kurtosis did not exceed 1.328, which does not indicate severe non-normality under Kline’s (2016) broad screening guidance. Spearman correlations, bootstrap intervals, and HC3 standard errors were retained as robustness checks for distributional and sampling concerns. For the main inferential summaries, two-sided tests used *p* < 0.05 as a conventional reference point. This threshold was not treated as a boundary between scientifically important and unimportant findings. Exact *p* values, standardized coefficients, confidence intervals, R^2^ and ΔR^2^, consistency across robustness checks, and theoretical plausibility were considered together, in line with guidance against interpreting *p* values as stand-alone measures of effect size, importance, or evidential certainty (Wasserstein & Lazar, 2016).

A diagnostic CFA treated the 50 focal items as four correlated first-order factors (EE-core, ROA, ESE, and EI). Fit was poor relative to commonly cited reference values (CFI = 0.583, TLI = 0.563, RMSEA = 0.103, SRMR = 0.092); the corresponding one-factor model fit substantially worse (CFI = 0.299, TLI = 0.270, RMSEA = 0.133, SRMR = 0.146; Hu & Bentler, 1999). These reference values were used diagnostically rather than as mechanical pass/fail cutoffs. The results do not support a well-fitting latent measurement model, so the aggregate specification is treated as exploratory and measurement-aware rather than as confirmatory evidence that the constructs are fully independent.

To support computational transparency, every focal score was reconstructed from respondent-level items using the explicit non-overlapping scoring rules described above. The composite definitions, standardization sequence, 10,000-resample bootstrap procedure, HC3 robustness checks, and diagnostic specifications are reported explicitly to provide an audit trail for re-analysis of the same analytic data. This level of documentation supports analytic transparency and facilitates computational re-analysis, while independent-sample replication remains a task for future research.

## 3. Results

### 3.1. Descriptive Statistics, Reliability, and Correlations

Table 2 reports descriptive statistics and reliability for the reconstructed indices. Cronbach’s α ranged from 0.846 to 0.921 and McDonald’s ω from 0.848 to 0.924. Their close agreement supports stable internal consistency estimates without implying that reliability alone establishes construct validity (Dunn et al., 2014). Neither skewness nor excess kurtosis indicated severe non-normality under Kline’s (2016) broad screening guidance.

Table 3 reports the Pearson correlations. EE-core and ROA were strongly correlated (r = 0.641, *p* < 0.01), requiring conservative interpretation. EI was positively correlated with EE-core (r = 0.231, *p* < 0.01), ROA (r = 0.241, *p* < 0.01), and ESE (r = 0.304, *p* < 0.01). Item-level HTMT was 0.750 for EE-core versus ROA and ranged from 0.217 to 0.332 for the remaining construct pairs, below the 0.85 reference proposed by Henseler et al. (2015). This pattern suggests some empirical separation, but the high EE-core-ROA correlation and suboptimal CFA mean that clear latent separation among all four constructs cannot be claimed.

### 3.2. Path Analysis and Bootstrap Tests

**Figure 2:**
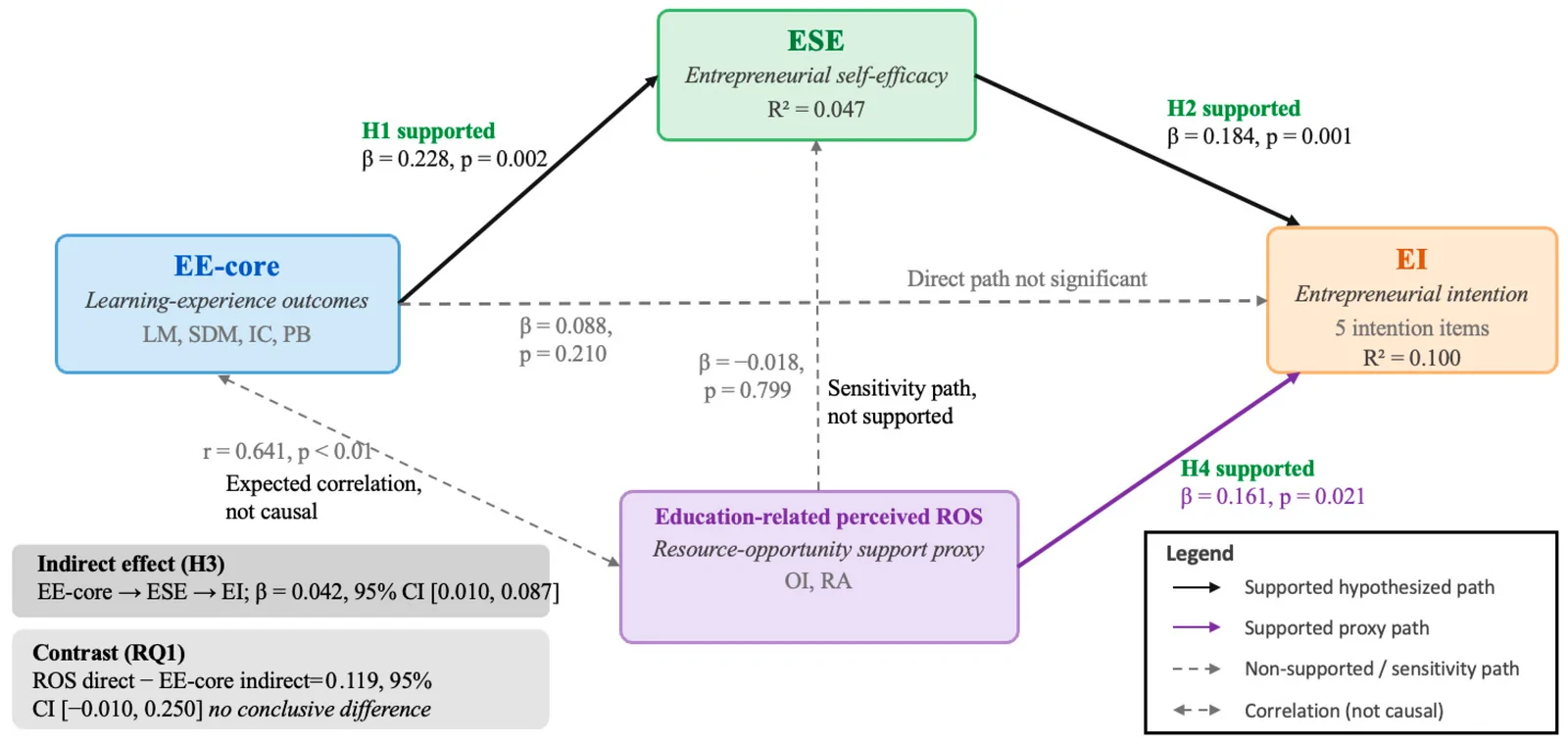
Empirical standardized estimates for the partial SCCT-informed composite model.

Table 4 reports standardized regression paths, the bootstrap indirect association, and the exploratory direct-versus-indirect contrast. EE-core was positively associated with ESE (β = 0.257, *p* < 0.001), whereas ROA showed little evidence of an additional association with ESE once EE-core was included (β = 0.029, *p* = 0.684); the ESE model explained 7.7% of the variance. Figure 2 visualizes the standardized estimates and the exploratory contrast.

In the EI model, ESE (β = 0.257, *p* < 0.001) and ROA (β = 0.150, *p* = 0.028) were positively associated with EI, whereas the direct EE-core estimate did not show a clear additional association (β = 0.064, *p* = 0.359). The model explained 12.9% of the variance in EI. The EE-core-ESE-EI indirect association was positive but small (β = 0.066, 95% bootstrap CI [0.025, 0.121]). The exploratory contrast between the ROA direct estimate and the EE-core-ESE-EI indirect estimate was 0.084 with a 95% CI of [−0.044, 0.213], providing no clear evidence that one route was larger than the other.

### 3.3. Robustness and Common Method Checks

Predictor VIF values were 1.768 for EE-core, 1.697 for ROA, and 1.083 for ESE. Full collinearity VIF values ranged from 1.148 to 1.773, below Kock’s (2015) 3.3 heuristic. These diagnostics reduce concern about unstable coefficients due to collinearity, but they do not establish construct independence or causal exogeneity.

Common method concerns were examined procedurally and statistically following recommendations for survey-based behavioral research (Podsakoff et al., 2003). Procedural safeguards included anonymity, voluntary participation, neutral wording, and data screening. The first unrotated component accounted for 19.74% of all questionnaire-item variance and 21.88% of the variance across the 50 focal items. The one-factor CFA fit substantially worse than the four-factor model (one-factor CFI = 0.299, TLI = 0.270, RMSEA = 0.133, SRMR = 0.146; four-factor CFI = 0.583, TLI = 0.563, RMSEA = 0.103, SRMR = 0.092). Because the four-factor model itself fit poorly relative to commonly used reference values (Hu & Bentler, 1999), these analyses are diagnostic only and cannot eliminate common-source bias, demonstrate exogeneity, or establish causality.

A controlled robustness regression was also estimated for EI (Table 5). Gender and grade alone explained little variance (R^2^ = 0.004). Adding EE-core and ESE increased R^2^ to 0.123; adding ROA increased it to 0.135 (ΔR^2^ = 0.012), and ROA remained positive (β = 0.143, *p* = 0.039). HC3 standard errors supported the same substantive conclusions for EE-core-ESE, ESE-EI, and ROA-EI. A sensitivity analysis using the full EIQ domains also produced positive ROA and ESE estimates, but it was not a primary test because that outcome combines intention with TPB antecedents. The results of H1–H4 are summarized in Table 6.

## 4. Discussion

The discussion considers the four hypotheses in turn. All four were consistent with the observed pattern of associations, but the effects and explained variance were modest. The EI model accounted for 12.9% of the variance and adding ROA to the controlled model increased R^2^ by 0.012. The findings are therefore best read as theoretically relevant associations, not as strong prediction or evidence of causal mechanisms.

### 4.1. EE-Core Perceived Competence and Entrepreneurial Self-Efficacy (H1)

The H1 result was consistent with the proposed relationship: EE-core perceived competence was positively associated with ESE (β = 0.257, *p* < 0.001). This pattern fits SCCT, in which mastery-related experiences, feedback, vicarious learning, and social persuasion contribute to efficacy beliefs (Bandura, 1997; Lent & Brown, 2019; Lent et al., 1994). It also aligns with entrepreneurship-education research linking education- or competence-related development with stronger entrepreneurial self-efficacy (X. Liu et al., 2019; X.-H. Wang et al., 2023; Wardana et al., 2020; Wu et al., 2022).

This result should be interpreted in light of how EE-core was measured. EE-core is a self-reported competence composite rather than a direct measure of course exposure, instructional intensity, or demonstrated performance. The H1 estimate therefore does not show that a particular EE intervention caused ESE to increase. A more defensible interpretation is that students who rated themselves more highly on the non-overlapping competence domains also tended to report greater confidence in performing entrepreneurial tasks.

### 4.2. Entrepreneurial Self-Efficacy, Intention, and the Indirect Association (H2–H3)

ESE was positively associated with EI (β = 0.257, *p* < 0.001), consistent with H2. Prior systematic reviews and empirical studies likewise show that efficacy beliefs are related to entrepreneurial intention because perceived capability contributes to whether entrepreneurship is seen as feasible and actionable (Newman et al., 2019; Piperopoulos & Dimov, 2015; Shahab et al., 2019; X.-H. Wang et al., 2023; Wu et al., 2022). In a vocational setting, this relationship is especially plausible because career goals are closely tied to perceived ability to perform applied tasks rather than to abstract preference alone.

The indirect association proposed in H3 was also positive but small (β = 0.066, 95% bootstrap CI [0.025, 0.121]). This pattern is compatible with entrepreneurship-education studies that position self-efficacy as one explanatory link between education-related experiences and entrepreneurial intention (Piperopoulos & Dimov, 2015; X.-H. Wang et al., 2023; Wu et al., 2022). It should not, however, be read as a demonstrated mediation process. All focal variables were measured at one time from the same respondents, so temporal ordering, reverse direction, and omitted-variable explanations remain plausible. ESE is therefore best viewed as one correlate in a wider intention-formation process rather than as a dominant causal mechanism.

### 4.3. Resource-Opportunity Accessibility and Entrepreneurial Intention (H4 and Exploratory Contrast)

ROA remained positively associated with EI after EE-core and ESE were included (β = 0.150, *p* = 0.028), consistent with H4. In the controlled robustness model, ROA remained positive (β = 0.143, *p* = 0.039) and increased R^2^ from 0.123 to 0.135 (ΔR^2^ = 0.012). Its incremental contribution is therefore statistically detectable but modest in practical terms. This pattern is compatible with research showing that perceived university support, ecosystem conditions, and opportunity-related learning can shape whether students view entrepreneurship as actionable (Elnadi & Gheith, 2021; Karimi et al., 2016; Saeed et al., 2015; Shirokova et al., 2016; Walter et al., 2013).

The Yiwu context provides a plausible substantive setting for this association because suppliers, market channels, logistics, digital platforms, and entrepreneurial role models are highly visible. Even so, the single-site design cannot show that Yiwu strengthens the ROA-EI relationship or that resource density acts as a moderator. More importantly, ROA is not a validated environmental-support measure. Its opportunity-identification and resource-acquisition items assess perceived capability and may only partly carry contextual-accessibility content. The strong EE-core-ROA correlation (r = 0.641) and the suboptimal four-factor CFA reinforce the need for this narrower interpretation.

The exploratory bootstrap contrast compared the ROA direct estimate with the EE-core-ESE-EI indirect estimate. The contrast was 0.084 with a 95% CI of [−0.044, 0.213], so the data did not provide clear evidence that the ROA route was stronger. Treating this comparison as exploratory rather than as a formal RQ is important because existing theory supports the relevance of both capability beliefs and contextual accessibility but does not provide a sufficiently strong basis for ranking their expected magnitudes. Taken together, the evidence is more consistent with both routes being relevant than with one clearly dominating the other.

### 4.4. Theoretical Implications

At the theoretical level, the study offers a measurement-focused, partial application of SCCT rather than a new environmental theory. SCCT is useful here because it accommodates learning-related outcomes, efficacy beliefs, contextual supports and barriers, and choice goals within one career-choice framework (Lent & Brown, 2019; Lent et al., 1994, 2000). The present model extends self-efficacy-centered entrepreneurship applications by placing ESE alongside a non-overlapping, competence-based ROA proxy while explicitly acknowledging that outcome expectations were not measured and that temporal ordering cannot be established.

The study also underscores the importance of construct discipline. Entrepreneurship-education scales can mix skill development, confidence, opportunity recognition, and resource acquisition. If the same item content is included both in an overall education score and in a separate contextual predictor, structural paths may partly reflect double counting. Using non-overlapping EE-core and ROA composites reduces this problem, while the proxy label prevents the resource/opportunity subdomain from being misrepresented as a newly validated environmental-support construct.

The modest R^2^ values are also informative. They show that EE-core, ESE, and ROA account for only part of the variation in entrepreneurial intention. Entrepreneurial mindset, personality traits (Vodă & Florea, 2019), family background, prior entrepreneurial exposure, institutional support, economic expectations, and SCCT outcome expectations may account for additional variance. Future comparative work should test whether the relative relevance of efficacy beliefs and contextual accessibility differs across resource-rich and resource-constrained settings rather than assuming that the Yiwu pattern generalizes automatically.

### 4.5. Practical Implications

For vocational educators, the findings suggest that EE design should combine competence development with guided access to resources and opportunities. Courses can pair business planning and task practice with opportunity-identification exercises, supplier or entrepreneur interaction, digital-commerce projects, mentoring, and structured use of incubation resources. Such activities are consistent with process-oriented and practice-based approaches to entrepreneurship education (Boldureanu et al., 2020; Karimi et al., 2016; Lyu et al., 2024).

For institutions in entrepreneurial regions, the practical issue is not simply whether resources exist but whether students can recognize, understand, and use them. In Yiwu, campus stores, e-commerce practice, cross-border trade training, mentors, local firms, and practice bases can be organized as structured learning opportunities. For policymakers, this implies that course availability should be considered alongside students’ ability to connect education with usable external support. Because the observed associations are modest and non-causal, these recommendations should be treated as design hypotheses for program development and evaluation rather than as demonstrated intervention effects.

## 5. Conclusions

This study examined how EE-core perceived competence, entrepreneurial self-efficacy, and a competence-based resource-opportunity accessibility proxy was associated with entrepreneurial intention among 322 students at one vocational college in Yiwu. The results were consistent with H1-H4: EE-core was positively associated with ESE (β = 0.257), ESE was positively associated with EI (β = 0.257), and the EE-core-ESE-EI indirect association was positive but small (β = 0.066, 95% bootstrap CI [0.025, 0.121]). ROA also remained positively associated with EI after EE-core and ESE were considered (β = 0.150). The exploratory direct-versus-indirect contrast did not provide clear evidence that the ROA route was stronger.

Conceptually, the study contributes a cautious, partial SCCT-informed account in which perceived competence, efficacy beliefs, and resource/opportunity accessibility are examined together without being treated as interchangeable constructs. Separating the entrepreneurial competence scale into non-overlapping EE-core and ROA composites reduces item double counting, while the ROA label makes clear that the resource/opportunity component is only a competence-based contextual-accessibility proxy. The findings therefore neither validate a stand-alone environmental-support construct nor show that contextual accessibility is more important than self-efficacy.

Practically, the findings suggest that entrepreneurship education in a resource-rich vocational setting may be more useful when it develops task confidence while also helping students recognize and navigate concrete opportunities, market channels, mentoring, suppliers, digital platforms, and incubation resources. The observed ΔR^2^ of 0.012 for ROA in the controlled model and the small indirect association through ESE indicate modest relationships rather than large program effects. Capability-building and guided resource access should therefore be treated as design priorities to be tested and evaluated, not as causal prescriptions established by the present data.

The study design also limits what can be inferred and generalized. All focal variables were self-reported by the same respondents at one time, so common-source covariance, response style, reverse direction, and endogeneity remain plausible. Unmeasured entrepreneurial mindset, personality, family background, prior entrepreneurial exposure, institutional support, economic expectations, and SCCT outcome expectations may confound the estimates. ROA was derived from competence items rather than a dedicated environmental-support scale, the four-factor CFA fit was suboptimal, and the single-institution sample should not be statistically generalized to other vocational colleges or regions. The documented scoring rules, analytic procedures, bootstrap settings, and robustness checks improve computational transparency, although independent replication requires new samples. Future longitudinal, multi-source, quasi-experimental, and comparative studies should measure EE exposure, outcome expectations, direct contextual support, baseline intention, and subsequent entrepreneurial behavior across vocational colleges, universities, Chinese regions, and international entrepreneurial ecosystems.

## Figures and Tables

**Table 1:** Construct specification and construct boundaries.

Construct	SCCT Role	Operationalization	Construct Rationale
EE-core	Education-related perceived competence outcome	Leadership and management; strategic decision-making; innovation and creativity; pressure bearing	Removes opportunity and resource dimensions from the education core to avoid overlap.
ROA proxy	Competence-based contextual-accessibility proxy	Opportunity identification and resource acquisition dimensions	Competence-based; not a validated environmental-support scale.
Entrepreneurial self-efficacy	Self-efficacy belief	De Noble et al. 23-item ESE scale	Retains established self-efficacy mechanism; not demonstrated competence.
Entrepreneurial intention	Choice goal	Five intention items from Liñán and Chen’s EIQ	Avoids combining TPB antecedents with intention; not observed behavior.

**Table 2:** Descriptive statistics and reliability.

Variable	Items	Mean	SD	α/ω	Skew	Kurtosis
EE-core	14	4.391	0.972	0.871/0.874	−0.021	−1.143
ROA proxy	8	4.330	1.085	0.846/0.848	−0.310	−0.884
Entrepreneurial self-efficacy	23	4.490	0.927	0.921/0.924	−0.183	−1.328
Entrepreneurial intention	5	4.096	1.367	0.897/0.900	−0.268	−1.005

Notes. N = 322. α = Cronbach’s alpha; ω = McDonald’s omega total.

**Table 3:** Pearson correlations among the reconstructed aggregate indices.

Variable	1	2	3	4
1. EE-core	1			
2. ROA proxy	0.641 **	1		
3. Entrepreneurial self-efficacy	0.276 **	0.194 **	1	
4. Entrepreneurial intention	0.231 **	0.241 **	0.304 **	1

Notes. ** *p* < 0.01 are conventional descriptive markers for the correlation matrix.

**Table 4:** Standardized path estimates and bootstrap tests.

Association	Estimate	SE	*p*	95% CI	Result
EE-core → ESE	0.257	0.070	<0.001	[0.120, 0.395]	Consistent with H1
ROA → ESE	0.029	0.070	0.684	[−0.109, 0.166]	No clear additional association
ESE → EI	0.257	0.054	<0.001	[0.150, 0.364]	Consistent with H2
ROA → EI	0.150	0.068	0.028	[0.016, 0.284]	Consistent with H4
EE-core → EI	0.064	0.070	0.359	[−0.073, 0.201]	No clear direct association
EE-core → ESE → EI	0.066	--	--	[0.025, 0.121]	Consistent with H3
Exploratory contrast: ROA direct − EE-core-ESE-EI indirect	0.084	--	--	[−0.044, 0.213]	No clear difference

Notes. Estimates are standardized. Bootstrap confidence intervals for the indirect association and exploratory contrast are based on 10,000 case-level resamples.

**Table 5:** Controlled robustness regression predicting entrepreneurial intention.

Predictor	Model 1: Controls Only	Model 2: EE-Core and ESE	Model 3: ROA
Gender (2 vs. 1)	−0.041 (*p* = 0.472)	−0.064 (*p* = 0.241)	−0.058 (*p* = 0.289)
Grade 2 vs. Grade 1	0.053 (*p* = 0.426)	0.071 (*p* = 0.259)	0.067 (*p* = 0.284)
Grade 3 vs. Grade 1	0.066 (*p* = 0.317)	0.069 (*p* = 0.267)	0.055 (*p* = 0.375)
EE-core	--	0.159 (*p* = 0.005)	0.069 (*p* = 0.325)
ESE	--	0.266 (*p* < 0.001)	0.262 (*p* < 0.001)
ROA	--	--	0.143 (*p* = 0.039)
R^2^	0.004	0.123	0.135
Adjusted R^2^	−0.005	0.109	0.118

Notes. Standardized coefficients are reported. Gender and grade are included as recorded in the dataset; Grade 1 is the reference category.

**Table 6:** Hypothesis summary.

Hypothesis	Tested Association	Result	Conclusion
H1	EE-core → ESE	β = 0.257, *p* < 0.001	Consistent with H1
H2	ESE → EI	β = 0.257, *p* < 0.001	Consistent with H2
H3	EE-core → ESE → EI	β = 0.066, 95% bootstrap CI [0.025, 0.121]	Consistent with H3; small indirect association
H4	ROA → EI after EE-core and ESE	β = 0.150, *p* = 0.028	Consistent with H4

## Data Availability

The participant-level data are available from the corresponding author upon reasonable request. The data are not publicly available because the original ethics and informed-consent arrangements did not include unrestricted public deposition of participant-level data, and the single-institution dataset presents a residual risk of deductive disclosure. Any data sharing will therefore be subject to applicable ethical and data-use requirements.
